# Economic Impact of Targeted and Immunotherapies in Treating Operable Esophageal and Non-Small Cell Lung Cancers

**DOI:** 10.1016/j.atssr.2025.01.008

**Published:** 2025-02-05

**Authors:** Yahya Alwatari, Mohamed Shanshal, Sameh Taki Aldin, Nate C. Johnson, Viengneesee Thao, Bijan J. Borah, K. Robert Shen

**Affiliations:** 1 Division of General Thoracic Surgery, Mayo Clinic, Rochester, Minnesota; 2 Division of Thoracic Medical Oncology, Mayo Clinic, Rochester, Minnesota; 3 Robert D. and Patricia E. Kern Center for the Science of Health Care Delivery, Mayo Clinic, Rochester, Minnesota

## Abstract

**Background:**

As the therapeutic landscape evolves, we aim to evaluate the cost implications of National Comprehensive Cancer Network–recommended perioperative targeted/immunotherapies for non-small cell lung cancers (NSCLCs) and esophageal cancers.

**Methods:**

The Medicare Part B payment allowance limits of treatment were ascertained. Using published data, we estimated the annual incidence of eligible patients. We applied the estimated cost of the medication based on treatment dosing and duration. The costs per patient and incident cohort were calculated.

**Results:**

We estimated that 8602 patients with newly diagnosed esophageal cancers would be eligible for adjuvant nivolumab. The cost to treat 1 patient was $190,000, and the cost to treat 1 incident cohort was $1.6 billion. We estimated that 50,409 patients with NSCLC will meet the criteria for neoadjuvant nivolumab with a cost of 3 cycles of $32,000 per patient and $1.7 billion per cohort. Among NSCLC patients who may undergo resection and qualify for adjuvant therapy, 70,602 patients are anticipated to be epidermal growth factor receptor–negative and treated with adjuvant atezolizumab or pembrolizumab. Treatment costs range from $178,000 to $197,000 per patient, with up to $13.9 billion cost per cohort. The cost to treat 1 patient with adjuvant osimertinib was $556,000, with an incident cohort cost of $8 billion. The cost to treat an incident cohort of eligible thoracic malignancies is estimated at $25 billion.

**Conclusions:**

Immune and targeted therapy in operable thoracic patients is associated with a significant cost burden. Studies are needed to assess cost-effectiveness to ensure optimal resource allocation and improve patient outcomes.

## Visual Abstract

**Figure:**
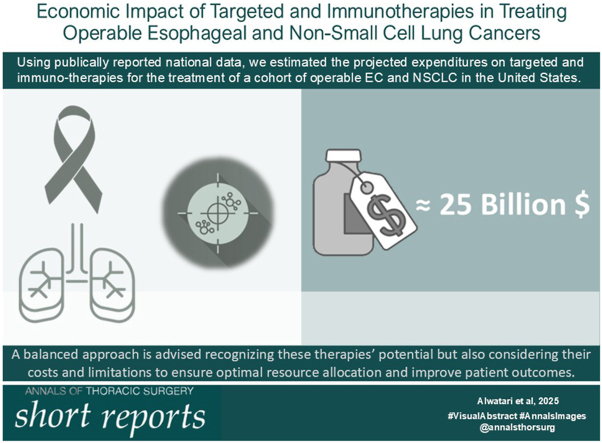

**In Short**

▪ Immune and targeted therapy in thoracic cancer patients is associated with a tremendous cost.
▪ Dedicated studies are timely needed to assess the cost-effectiveness of emerging therapies.
▪ A balanced approach is advised recognizing their potential but considering their costs and limitations.

In 2024, the United States is expected to see >234,000 new lung cancer cases and ∼125,000 deaths. Esophageal cancer (EC) will have >22,000 new cases and >16,000 deaths.^1^ Lung cancer remains the leading cause of cancer-related deaths, and EC is among the deadliest gastrointestinal cancers. Immunotherapy and targeted therapy have revolutionized thoracic cancer treatment and are increasingly integrated into the guidelines of leading societies. Although these therapies offer disease-free survival benefits for NSCLC and EC, several lack clear evidence of overall survival improvement. A study by Gyawali and colleagues^2^ found that 20% of cancer drug indications approved by the United States Food and Drug Administration’s accelerated pathway showed overall survival benefits in confirmatory trials.

The National Comprehensive Cancer Network (NCCN) recommends atezolizumab, nivolumab, osimertinib, and pembrolizumab for perioperative management of NSCLC and ECs. These therapies offer benefits, but their high costs can cause financial hardship, leading patients to forego or delay treatment. This study was conducted to fill a gap in understanding the cost implications of immune/targeted therapies in operable EC and NSCLC cohorts. Policymakers can use these results to shape health care policies and reimbursement models and to make informed decisions on insurance coverage, drug pricing, and patient support programs, ensuring equitable health care.

## Patients and Methods

### Patient Eligibility

**Figure 1:**
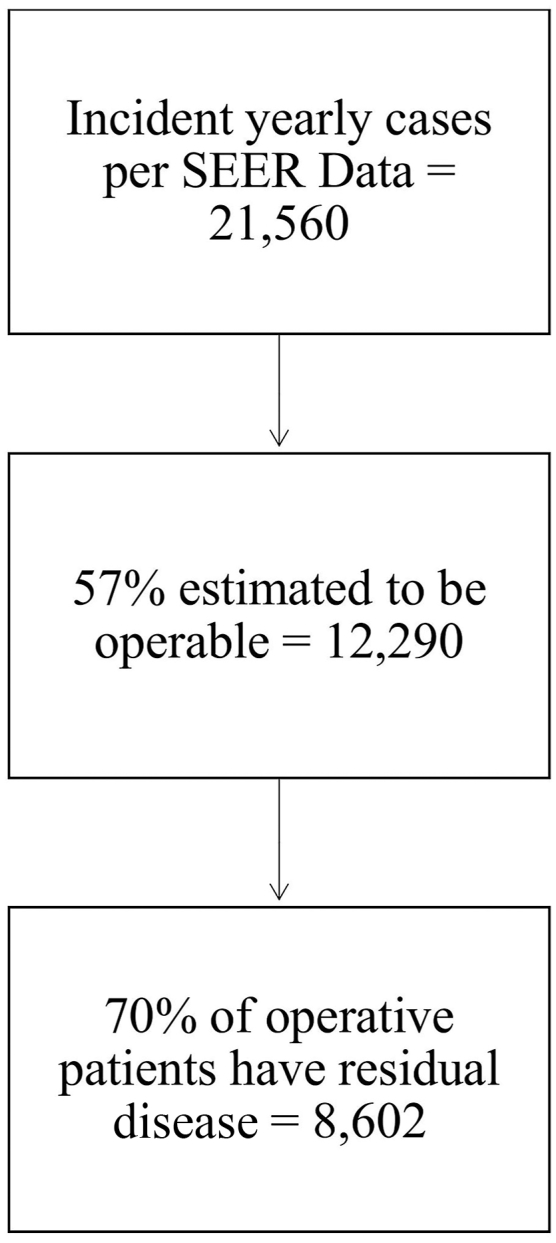
Estimation of the number of operative esophageal cancer patients with eligibility for adjuvant nivolumab. (SEER, Surveillance, Epidemiology, and End Results.)

**Figure 2:**
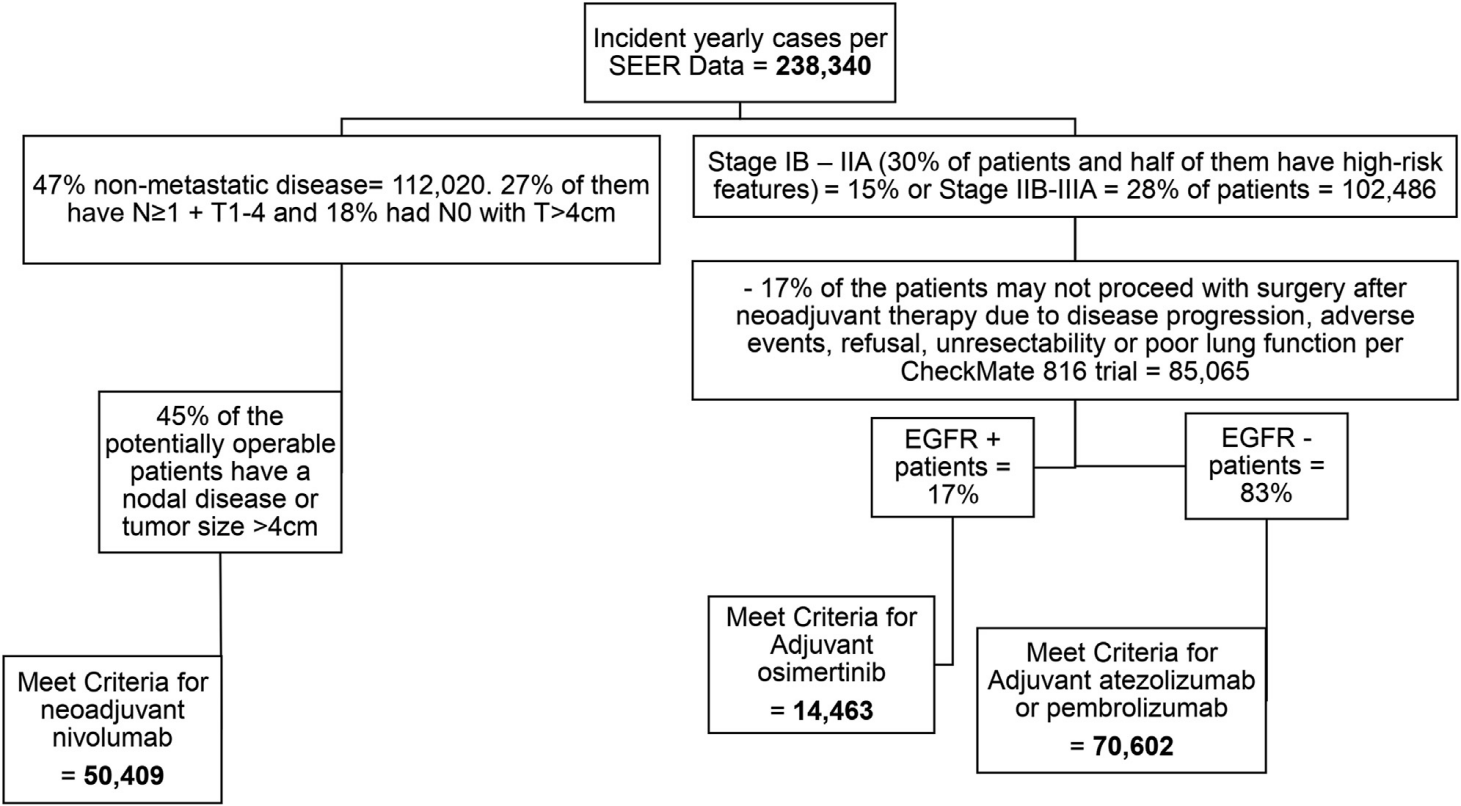
Estimation of the number of operative non-small cell lung cancer (NSCLC) patients with eligibility for neoadjuvant and/or adjuvant immune and targeted therapies. (CheckMate 816, A Neoadjuvant Study of Nivolumab Plus Ipilimumab or Nivolumab Plus Chemotherapy Versus Chemotherapy Alone in Early Stage Non-Small Cell Lung Cancer [NSCLC]; EGFR, epidermal growth factor receptor; SEER, Surveillance, Epidemiology, and End Results.)

Using national data and literature, we estimated the annual incidence of newly diagnosed EC and NSCLC patients eligible for surgery with neoadjuvant and/or adjuvant immune and targeted therapy according to NCCN guidelines. For EC, we used Surveillance, Epidemiology, and End Results (SEER) data and literature on operable cases and postresection residual disease to estimate adjuvant nivolumab eligibility (Figure 1). To estimate the number of operative NSCLC patients eligible for neoadjuvant and/or adjuvant immune and targeted therapies, we used SEER data, TNM classification, and literature on targeted mutations (Figure 2). Patients with advanced inoperable cancers were excluded due to the complexity of their care.

### Costs Calculation

Costs were defined as direct episode payments, which included drug acquisition costs and management of adverse events (AEs). We determined the Medicare Part B payment limits for each medication, except osimertinib, for which we used the wholesale acquisition cost due to Medicare Part D coverage. Costs were based on dosing and treatment duration, typically 1 year, except for adjuvant osimertinib, which spanned 3 years. Using these data, we estimated the cost per patient and the total cost for an incident cohort. Results are presented as the cost per patient and the total cost to Medicare for treating 1 cohort of NSCLC and EC patients.

### AE Costs Estimation

We used patient-level data from trials on atezolizumab, nivolumab, osimertinib, and pembrolizumab to estimate rates of grade 3/4 AEs in eligible EC and NSCLC patients. The analysis included patients who received at least 1 dose of the treatments. The Healthcare Cost and Utilization Project (HCUP) National Inpatient Database provided the unit cost of each grade 3/4 AE.

Developed through a federal-state-industry partnership, the HCUP database is a family of health care databases with a national information resource of encounter-level health care data. The Common Terminology Criteria for Adverse Events defines patients with grade 3 AEs as those potentially requiring hospitalization and patients with grade 4 AEs as those requiring urgent intervention; therefore, the costs from the HCUP database should be representative to estimate the unit costs associated with each AE.^3^ The total potential costs were calculated by multiple percentages of grade 3/4 AEs by eligible cohorts by reported costs.

The Checklist and Explanation and Elaboration Task Force Report 2022 statement for health economic evaluation was reported (Supplemental Consolidated Health Economic Evaluation Reporting Standards [CHEERS] Checklist).

## Results

### Eligible Patients and Recommended Therapies

Supplemental Table 1 outlines NCCN recommendations for immune and targeted therapies for operable NSCLC and EC patients. For operable EC with residual disease after resection, nivolumab, 240 mg intravenous every 14 days for 16 weeks, then 480 mg every 28 days for 1 year, is recommended (An Investigational Immuno-therapy Study of Nivolumab or Placebo in Participants With Resected Esophageal or Gastroesophageal Junction Cancer [CheckMate 577]).

For neoadjuvant therapy in NSCLC, patients with tumors ≥4 cm or node-positive should receive nivolumab, 360 mg, and platinum-doublet chemotherapy every 3 weeks for 3 cycles (A Neoadjuvant Study of Nivolumab Plus Ipilimumab or Nivolumab Plus Chemotherapy Versus Chemotherapy Alone in Early Stage Non-Small Cell Lung Cancer [NSCLC] [CheckMate 816]).

In the adjuvant setting for operable NSCLC, stage IB to IIIA patients with high-risk features should receive chemotherapy, followed by atezolizumab, pembrolizumab, or osimertinib. High-risk features include poorly differentiated tumors, vascular invasion, wedge resection, visceral pleural involvement, and unknown lymph node status. Recommended therapies are osimertinib, 80 mg daily, for 3 years (AZD9291 Versus Placebo in Patients With Stage IB-IIIA Non-small Cell Lung Carcinoma, Following Complete Tumour Resection With or Without Adjuvant Chemotherapy [ADAURA]), atezolizumab, 1200 mg every 3 weeks for up to 1 year (Study to Assess Safety and Efficacy of Atezolizumab [MPDL3280A] Compared to Best Supportive Care Following Chemotherapy in Patients With Lung Cancer [IMpower010]), and pembrolizumab, 200 mg every 3 weeks for up to 1 year (Study of Pembrolizumab (MK-3475) vs Placebo for Participants With Non-small Cell Lung Cancer After Resection With or Without Standard Adjuvant Therapy [MK-3475-091/KEYNOTE-091] [PEARLS]).

### Total Costs of Treatment

As determined by the mathematical model, ∼8600 patients with EC are expected to be eligible for surgery and adjuvant nivolumab yearly. The calculations are based on an estimated 21,560 new cases in 2023 of EC from these SEER data, with 57% being locoregional and eligible for resection. De Gouw and colleagues^4^ reported in a systematic review and meta-analysis that approximately two-thirds of patients will have residual disease after resection, making them eligible for nivolumab. The estimated total cost per average Medicare payment is ∼$7300 per dose for the first half of the therapy and ∼$14,600 for the second half, with a total cost of $190,000 per patient and $1.6 billion per cohort of eligible EC patients (Supplemental Table 2; Table 1).

**Table 1:** Cost of Targeted and Immunotherapies in Operable Non-Small Cell Lung Cancer and Esophageal Cancer Cohort

Cancer	Therapy	Cost per Person ($)	Incident Cases (n)	Cost to Medicare to Treat Incident Cohort ($)
Esophagus	Adjuvant nivolumab	190,052	8602	1,634,824,551
NSCLC	Neoadjuvant nivolumab	32,894	50,409	1,658,131,466
NSCLC (non-EGFR)	Adjuvant atezolizumab or pembrolizumab	$178,584-197,320	70,602	12,608,387,568-13,931,186,640
NSCLC (EGFR+)	Adjuvant osimertinib	556,720^a^	14,463	8,051,841,360
Total cost, $	23,953,184,945-25,275,984,017			

EGFR, epidermal growth factor receptor; NSCLC, non-small cell lung cancer.

a Discount rate 3% per year applied.

For NSCLC, the same calculation methodology was applied. SEER data showed 238,340 new cases of lung cancer in 2023, with 53% being nonoperable secondary to distant metastasis. The distribution of T, N, and M categorization in the staging population proposed by the International Association for the Study of Lung Cancer (Lung Cancer Staging Project) Proposals for Revision of the TNM Stage Groupings in the Eighth Edition of the TNM Classification for Lung Cancer were used to calculate the numbers of eligible patients.^5^

In addition to that, the estimated prevalence of molecular aberrations in patients with NSCLC from the Hirsch and colleagues^6^ published report in *The Lancet* was taken into account (Figure 2). The estimated cost of neoadjuvant nivolumab was $32,000 with $1.65 billion per cohort. Atezolizumab cost per patient was ∼$179,000, whereas pembrolizumab was $197,000. The cost per their respective cohort was between $12 and $13.9 billion. Adjuvant osimertinib per patient cost was the highest at $573,000 with almost $8 billion per patient cohort. The estimated total cost was ∼$25 billion (Table 1).

### Costs of AEs

The rates of grade 3 to 4 AEs ranged from 11% to 20% per reciprocal trial, with treatment costs averaging $11,450 (range, $7400-$20,200). The total cost for grade ≥3 AEs was $3.2 million for adjuvant nivolumab in EC and $28.5 million for neoadjuvant nivolumab in NSCLC. Adjuvant therapy costs reached $60 million for atezolizumab, $82.8 million for pembrolizumab, and $18 million for osimertinib (Supplemental Table 3). The total estimated costs for the EC and NSCLC cohorts ranged from $110 million to $133 million (Table 2).

**Table 2:** Potential Cost Related to Treatment of Grade ≥3 Medication-Related Adverse Events

Cancer	Therapy	Rate of Grade ≥ 3 AEs	Incident Cases (n)	Cost to Medicare to Treat Incident Cohort ($)
Esophagus	Nivolumab	0.13	8,602	3,281,376
NSCLC	Nivolumab^a^	0.18	50,409	28,538,857
NSCLC (non-EGFR)	Atezolizumab or pembrolizumab	0.11 0.15	70,602	60,481,666 or 82,862,524
NSCLC (EGFR+)	Osimertinib	0.20	14,463	18,371,314
Total cost, $	110,673,213-133,054,071			

EGFR, epidermal growth factor receptor; NSCLC, non-small cell lung cancer.

a Neoadjuvant therapy.

## Comment

Our study estimated the total treatment cost for a cohort of operable EC and NSCLC patients using a mathematical model based on NCCN guidelines and national data. The costs could exceed $25 billion, posing a significant burden on the American health care system. This cost is expected to rise with the Food and Drug Administration approval of more therapies. For comparison, hemodialysis care, which is one of the most expensive national expenditures, costs Medicare ∼7% of its budget and nearly 1% of the entire federal budget, totaling $28 billion.^7^ The potential cost from our study is double Singapore’s 2021 health sector budget ($11 billion US) and 80% of the EU’s 2018 cancer drug expenditure.^8^

In 2021, US health spending reached $4.3 trillion, 18.3% of Gross Domestic Product, with $13,439 spent per capita in 2022. The cost from our study equals what the US pays to treat 2.2 million patients yearly. The US spends more on health care than any other nation, yet faces challenges in achieving comparable health outcomes, including lower life expectancy.^9^

Assessing the cost-effectiveness of perioperative immune and targeted therapy is complex. Factors such as therapeutic indication, patient preference, comorbidities, and biomarkers impact cost-effectiveness. Selecting appropriate candidates is crucial to optimize outcomes and resource use. Subgroup analysis, predictive biomarkers, and artificial intelligence models can enhance treatment efficacy and personalize care. Developing best practice algorithms helps limit overtreatment and financial toxicity.^10^

Our calculated analysis of the cost of hospitalization, defined as the development of grade ≥3 AEs, reveals significant variations across different cancer therapies (Table 2). The estimated cumulative Medicare expenditure for managing grade ≥3 AEs among incident patient cohorts ranges from $110 million to $133 million. These findings underscore the necessity of careful consideration regarding the cost implications of hospitalization associated with various cancer therapies.^11^

Our study has limitations, including assuming full access to standard care and not accounting for medication price variations between health care systems or insurance types. We used Medicare data for its wide availability and standardized approach. We did not calculate the cost of delivering medications or treating grade 1 and 2 AEs or evaluate patient intolerance or dose reductions. We used the rate of complications related to treatment as reported in the original trials, which may differ when applied to a larger population.

The study does not address cost-effectiveness or cost comparison between medical and surgical therapies, which involves quality-adjusted life years and incremental cost-effectiveness ratios. However, it uses established oncology guidelines and national data for cost calculations. Balancing costly therapeutics with the financial well-being of the health care system is crucial for equity. Large-scale studies on cost-effectiveness are needed to guide policy decisions and guidelines. It is important to recognize that the field is evolving and that future estimates may change as additional trials are conducted and the tumor genetics are better understood.
