# Supplementary material for: Economic Impact of Targeted and Immunotherapies in Treating Operable Esophageal and Non-Small Cell Lung Cancers
Source: Ann Thorac Surg Short Rep. 2025 Feb 5;3(4):1129–34. doi: 10.1016/j.atssr.2025.01.008 (PMC12711689; doi:10.1016/j.atssr.2025.01.008)
Supplement: Supplementary Table 1 [file mmc2.docx]

**Supplemental Table 1**. NCCN Recommendations for medications dose and duration for treatment of eligible operable NSCLC and EC patients. In Bold, the regimen was utilized per the original randomized controlled trial.

| **Cancer** | **Drug (Trial)** | **Dose (mg)** | **Duration of Adjuvant Treatment** |
| --- | --- | --- | --- |
| **NSCLC** | Neoadjuvant nivolumab  (CheckMate 816) | 360 | **Every 3 weeks for 3 cycles** |
|  | Adjuvant atezolizumab  (IMpower010) | 840  1200  1680 | Every 2 weeks for 1 year = 26 times in a year or  **Every 3 weeks for 1 year = 18 times in a year** or  Every 4 weeks for 1 year = 13 times in a year |
|  | Adjuvant pembrolizumab  (PEARLS/KEYNOTE-091) | 200  400 | **Every 3 weeks for 1 year = 18 times in a year** or  Every 6 weeks for 1 year = 9 times in a year |
|  | Adjuvant osimertinib  (ADAURA) | 80 | **Daily for 3 years** |
| **EC** | Adjuvant nivolumab  (CheckMate 577) | 240  480 | **Every 14 days for 16 weeks = 8 times in a year**  **Every 28 days for 36 weeks = 9 times in a year** |
