# Supplementary material for: Economic Impact of Targeted and Immunotherapies in Treating Operable Esophageal and Non-Small Cell Lung Cancers
Source: Ann Thorac Surg Short Rep. 2025 Feb 5;3(4):1129–34. doi: 10.1016/j.atssr.2025.01.008 (PMC12711689; doi:10.1016/j.atssr.2025.01.008)
Supplement: Supplementary Table 2 [file mmc3.docx]

**Supplemental Table 2.** Cost of recommended neoadjuvant and adjuvant medications per dose with Average Whole Sale price and Medicare Payment Limit

| Cancer | Medication | Medicare payment limit (per mg) | AWP (Average Wholesale Price, per mg) | Medicare payment limit (as % of AWP) | mg/dose | Cost/dose ($) | Recommended Frequency/year | Cohort Treatment Cost |
| --- | --- | --- | --- | --- | --- | --- | --- | --- |
| **NSCLC** | nivolumab^*^ | 30.46 | 36.691 | 0.830 | 360 | 10964.52 | 3 | $32,893.56 |
|  | atezolizumab | 8.27 | 10.34717 | 0.799 | 1200 | 9921.36 | 18 | $178,584.48 |
|  | osimertinib | 6.54 | 8.067375 | 0.811^ϒ^ | 80 | 523.52 | 365 | $573,254.4 |
|  | pembrolizumab | 54.81 | 65.3828 | 0.838 | 200 | 10962.2 | 18 | $197,319.6 |
| **EC** | nivolumab | 30.46 | 36.691 | 0.830 | 240 | 7309.68 | 8 | $58,477.44 |
|  | nivolumab | 30.46 | 36.691 | 0.830 | 480 | 14619.36 | 9 | $131,574.24 |

^*^ Neoadjuvant therapy

^ϒ^ Estimation in comparison to other medications (Coverage under Medicare group D). Recommended therapy is for 3 years
